# Importance of increasing resection rates in pancreatic cancer treatment

**DOI:** 10.1093/bjsopen/zraf029

**Published:** 2025-03-25

**Authors:** Marco Del Chiaro, Hiroyuki Ishida, Richard D Schulick

**Affiliations:** Division of Surgical Oncology, Department of Surgery, University of Colorado School of Medicine, Aurora, Colorado, USA; Division of Surgical Oncology, Department of Surgery, University of Colorado School of Medicine, Aurora, Colorado, USA; Department of Hepatobiliary and Pancreatic Surgery, Institute of Science Tokyo, Tokyo, Japan; Division of Surgical Oncology, Department of Surgery, University of Colorado School of Medicine, Aurora, Colorado, USA

Even though surgery remains the only chance of cure, the development of new systemic therapies observed over the past decade has significantly contributed to the improvement of survival in patients with pancreatic cancer^1^. Recent advances in systemic chemotherapy, such as FOLFIRINOX (leucovorin calcium (folinic acid), fluorouracil, irinotecan hydrochloride, and oxaliplatin) and gemcitabine plus nab-paclitaxel, have expanded the pool of surgical candidates, particularly for patients with borderline resectable and locally advanced pancreatic cancer^2^. Conversion surgery has also emerged as a promising approach for selected patients with initially metastatic disease^3^. Consequently, resection rates have increased over time, particularly in high-volume centres that integrate modern chemotherapy with advanced surgical techniques. However, significant disparities in hospital resources, experience, and patient access globally underscore the need to understand the relationship between resection rates and survival outcomes at a population level.

The systematic review by Lockie *et al*. provides critical insights into these challenges, demonstrating a significant association between higher resection rates and improved survival^4^. By analysing data from 516 789 patients across 19 studies, the authors establish a robust relationship between resection rates and both 1-year survival and median overall survival. Their model predicts substantial survival benefits from increases in resection rates, even when accounting for potential increases in the perioperative mortality rate^4^. By quantifying the relationship between resection rates and the perioperative mortality rate and their impacts on survival, healthcare systems can optimize both treatment efficacy and patient safety.

Achieving higher resection rates requires multidisciplinary care. Multidisciplinary teams, including medical oncologists, radiologists, and surgeons are essential in refining patient selection for surgery through integrating biomarkers, radiological findings and technical feasibility. Neoadjuvant chemotherapy plays a pivotal role in borderline resectable and locally advanced pancreatic cancer, enabling preoperative systemic disease control and enhancing the survival benefit of surgery. Furthermore, centres of excellence with experienced pancreatic surgeons are crucial in managing complex cases, such as those requiring vascular resection and reconstruction, while ensuring robust perioperative rescue systems^5^. However, as the authors mentioned, access to these highly specialized centres remains a challenge for patients, due to socioeconomic, geographic and infrastructural barriers. Centralization and coordinated referral systems are therefore indispensable to ensure equitable access to these centres and to optimize patient outcomes globally.

Although the study by Lockie *et al*. provides valuable insights, several limitations should be acknowledged. The included studies span a relatively long time frame, with only 3 of 19 studies being after 2011. Therefore, the applicability of their findings to current treatment paradigms is limited. Although trends of increasing resection rates alongside higher use of chemotherapy and improved survival are observed, the population-level impact of modern chemotherapy regimens on resection rates and survival outcomes remains underexplored.

Despite these limitations, the study by Lockie *et al*. clearly highlights the importance of increasing resection rates in pancreatic cancer treatment. Establishing a benchmark resection rate would provide a framework for guiding equitable access to high-quality pancreatic cancer care and for evaluating regional healthcare system performance. In addition, centralization of care, standardization of the training for the new generation of pancreatic surgeons (that more often will deal with complex vascular resections and reconstructions), combined with efforts to reduce barriers to specialized treatment, are critical steps in improving pancreatic cancer survival. Further research is needed to evaluate the impact of modern chemotherapy regimens on resection rates and survival outcomes.
